# Perforation of the Terminal Ileum Secondary to Mucosal Damage of Enteroaggregative Escherichia coli and a Toothpick

**DOI:** 10.7759/cureus.62495

**Published:** 2024-06-16

**Authors:** Nora A Rady, James Parrish

**Affiliations:** 1 Research, Edward Via College of Osteopathic Medicine - Louisiana Campus, Monroe, USA; 2 Surgery, Christus St. Frances Cabrini Hospital, Alexandria, USA

**Keywords:** surgery, mucosal ulceration, intestinal obstruction, gastrointestinal perforation, gastroenteritis, foreign body, escherichia coli

## Abstract

Enteroaggregative *Escherichia coli* (EAEC) is a common form of *E. coli* that causes gastroenteritis and diarrhea worldwide. Biofilm formation on the intestinal mucosa initiates an inflammatory cascade in the gastrointestinal tissue, which has significant destructive effects on the mucosa of the small and large intestines. Small bowel obstruction and perforation due to a foreign body are uncommon, but the risk increases with pre-existing conditions such as the presence of intestinal strictures, inflammation, and mucosal ulceration. We present a unique case of acute enteritis from EAEC with mucosal ulceration and perforation because of co-ingestion of foreign body and impaction with the presence of stricture in the terminal ileum. This was treated with small bowel resection and primary anastomosis. The patient was successfully discharged from the hospital. The clinical features and pathological findings of enteric EAEC infection are described. To our knowledge, intestinal perforation and secondary peritonitis related to EAEC enteric infection, with mucosal ulceration and perforation secondary to co-ingestion of a foreign body with intestinal stricture, have not been documented. In this case, EAEC was associated with terminal ileum mucosal ulceration and complicated by perforation secondary to foreign body impaction along with ileal stricture. These compounding effects likely explain gastrointestinal tract perforation and secondary peritonitis.

## Introduction

Enteroaggregative *Escherichia coli* (EAEC) is a common form of bacterial gastroenteritis and diarrhea [1]. The symptoms include watery diarrhea with variable blood and mucus, nausea, and vomiting lasting from one to two weeks [2]. This strain utilizes aggregative adherence fimbriae and biofilm formation to attach to the intestinal mucosa and initiate an inflammatory cascade in the gastrointestinal tissue [1]. EAEC can destroy the mucosa of the small and large intestine via biofilm formation [2,3]. The biofilm is grossly characterized by a thick green-yellow mucus layer with a preference for the distal ileum and ascending colon [4]. Ciprofloxacin is generally used in the treatment of EAEC gastroenteritis [5].

Small bowel obstruction (SBO) and perforation due to foreign bodies are uncommon because 80-90% pass through the gastrointestinal tract without additional intervention, and only 15-35% of ingested sharp foreign bodies cause perforation [6]. The risk can increase with pre-existing conditions such as the presence of intestinal strictures, inflammation, and/or mucosal ulceration. Here we describe a unique case of acute EAEC enteric infection with ileal ulceration and co-ingestion of a foreign body impacted in an ileal stricture, causing perforation and secondary peritonitis, which to our knowledge has not been reported in the literature.

## Case presentation

A 66-year-old Caucasian male presented with a two-day history of pain in his left lower quadrant, with nausea, vomiting, and diarrhea, which was similar to previous abdominal pain he experienced during an episode of acute diverticulitis treated with partial colectomy 10 years ago. There were no fever or weight changes. Past medical history included chronic obstructive pulmonary disease, emphysema, diverticulitis, hypertension, anxiety, and depression.

Admission vital signs were a temperature of 36.4°C, pulse of 118 per minute, respiration of 16 per minute, and blood pressure of 115/88 mmHg. Abnormal laboratory values included an elevated white blood cell count of 16.7 × 10^9^/L, elevated serum creatinine of 1.43 mg/dL, low serum calcium of 6.7 mg/dL, and a low serum potassium level of 2.9 mEq/L. Stool PCR was positive for EAEC. No abnormalities were noted on urinalysis. Blood cultures were negative. Due to intolerance to oral contrast, an abdominal CT was performed with IV contrast only and demonstrated inflammation of a single loop of small bowel with small bubbles of extraluminal gas, and fat stranding of the mesentery. Inflammation was also present in the nearby bladder dome.

The patient was started on IV metronidazole and ciprofloxacin and a clear liquid diet. An initial diagnosis of acute EAEC gastroenteritis was made. Over the next two days, the patient was improving with IV antibiotics, hydration, and electrolyte replacement. Laboratory values showed improvement with creatinine at 0.76 mg/dL, serum potassium of 3.1 mEq/L, and white cell count of 9.5 × 10^9^/L. A plain abdominal radiograph on day 2 demonstrated dilated loops of small bowel possibly representing adynamic ileus.

On days 3 and 4, the patient had non-bilious and non-bloody emesis. The patient continued on a full liquid diet, and by day 4, laboratory values showed an elevated white blood cell count of 13.4 × 10^9^/L, a low serum phosphate level of 2.1 mg/dL, and an elevated blood pressure of 159/72 mmHg. The patient continued to be afebrile, with a normal pulse of 61 per minute and a respiratory rate of 18 per minute. Mild tenderness to palpation without rebound or guarding was found on abdominal examination. On day 4, a plain abdominal radiograph showed numerous dilated small bowel loops consistent with either adynamic ileus or SBO. A nasogastric (NG) tube was inserted to decompress the small bowel loops. Gastric drainage from the NG tube was 450 mL on day 4 and 950 mL on day 5.

**Figure 1:**
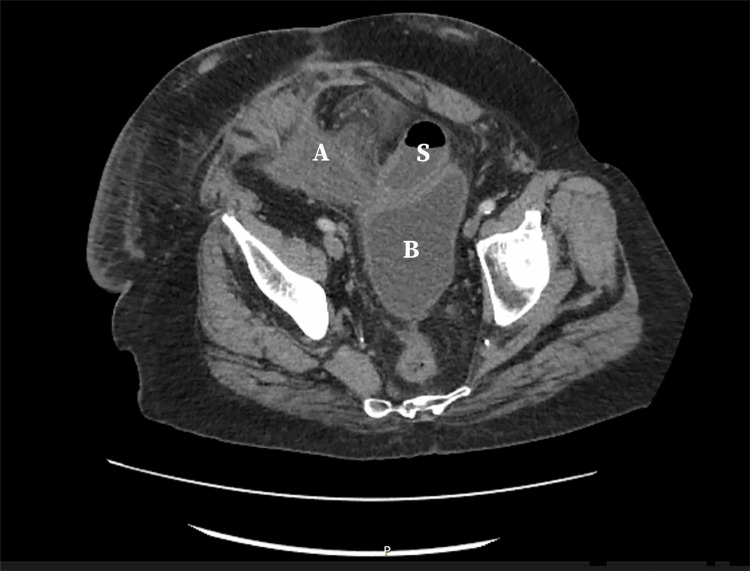
Abdominal computerized tomography Abdominal computerized tomography on day 5 reported inflammatory changes and a large phlegmon above the dome of the urinary bladder (B), with gas foci consistent with the formation of an extraluminal abscess (A), appearing low-attenuation, and significant small bowel distention (S) within the peritoneal cavity.

On day 5, abdominal computed tomography reported new findings, including a large phlegmon above the dome of the urinary bladder with several extraluminal low-attenuation and gas-containing foci suggesting abscess formation, as well as significant small bowel distention within the peritoneal cavity that was not previously observed. The patient was afebrile with a white blood cell count of 12.0 × 10^9^/L (Figure 1).

On day 6, exploratory laparotomy was performed due to a lack of clinical improvement. The findings at laparotomy included dilated small bowel loops and a linear foreign body palpable in an inflammatory area of the terminal ileum. The foreign body had protruded through the wall of the small bowel into the mesentery and was grossly consistent with a toothpick. The small bowel was transected proximally and distally through a healthy, noninflamed bowel, resulting in resection of a 32 cm loop of terminal ileum with primary anastomosis.

The gross pathological examination of the resected ileum revealed two areas of ulceration, each of which was 1.6-2.8 cm, one of which contained the area of perforation. The larger area of ulceration had green discoloration and a nearby edematous stricture. Beyond the stricture were sections of the bowel representative of inflammation. Microscopic findings revealed ulceration of mucosa associated with perforation and suppurative fibrinous visceroperitonitis.

The patient was started on total parenteral nutrition postoperatively, converted to a clear liquid diet on day 11 (postoperative day 5), and was advanced to a regular diet on day 13. The patient was discharged to inpatient rehabilitation on day 17 (postoperative day 11).

## Discussion

The initial admission diagnosis was consistent with infectious gastroenteritis due to symptoms, elevated white blood cell count, and positive stool PCR for EAEC. Subsequent presentation of protracted small bowel ileus and development of extra-luminal abscess indicated that infectious enteritis was likely complicated by mechanical obstruction and perforation of the small bowel. This was confirmed at laparotomy and pathologic examination of the resected small bowel revealed an impacted toothpick foreign body, stricture, and perforation through the wall of the small bowel.

The resected segment of the terminal ileum contained two areas of ulceration, one of which is stated to be an area where the perforation occurred. EAEC is known to cause mucosal ulceration in the terminal ileum through establishment in the gastrointestinal tract via biofilm formation [7]. Gross histopathologic examination indicated likely biofilm formation in the area of ulceration with green discoloration. The wall perforation in the terminal ileum likely occurred because of the impacted foreign body, a toothpick, in the area of mucosal ulceration in this patient. Also, histopathologic examination indicated the presence of edematous ileal stricture associated with the site of foreign body impaction and perforation. The majority of ingested foreign bodies usually pass through the gastrointestinal tract without causing mechanical obstruction or perforation of the small bowel [6]. However, the presence of ileal stricture may have prevented the passage of foreign bodies through the gastrointestinal tract and caused impaction in ulcerated mucosa of the terminal ileum and subsequent perforation.

EAEC and gastrointestinal tract 

EAEC creates conditions for necrosis of gastrointestinal epithelial tissue and mucosa ulceration. Mucinous biofilm formation in the gastrointestinal tract establishes the residence and is a defining feature of EAEC [7]. In the normal gastrointestinal lumen, the microbiome comprises the most superficial apical layer of mucosa, followed by a thick wall of mucus that acts as a protective layer above the gastrointestinal epithelial cells, which bacteria can only partially penetrate [1,8,9]. Transmembrane mucin receptors, namely Muc1, span the width of this mucinous layer into the lumen of the gastrointestinal tract [1,10]. Muc1 has been observed to help prevent infection by playing a role in mucosal defense and acting as a decoy receptor [1,9,11].

Muc1 can be hijacked to be used as a host-cell receptor for EAEC [1]. One of the primary virulence factors of EAEC, aggregative adherence fimbriae (AAF) adhesins bind to sialic acid residues on Muc1 receptors [1]. AAF is responsible for EAEC’s ability to adhere to the intestinal mucosa and biofilm formation [1,7]. The co-occurring inflammatory response in the gastrointestinal system elicited by EAEC is mediated by Muc1. This protein facilitates EAEC's ability to trigger the release of eicosanoid-based polymorphonuclear attractants in the gastrointestinal tract, promoting the migration of polymorphonuclear neutrophils through the intestinal epithelium [1,12]. When Muc1 is downregulated, EAEC’s recruitment of polymorphonuclear neutrophils is significantly reduced [1].

Gastrointestinal ingestion of foreign body

Foreign body perforation of the gastrointestinal tract is extremely uncommon as most foreign bodies can pass through spontaneously. Underlying conditions can create strictures and other abnormalities in the gastrointestinal tract, which prevent foreign bodies from passing through. The duodenum and sigmoid colon are common sites of foreign body perforation.

Perforation by toothpick of the stomach, ileum, and sigmoid colon has been documented. Presentation of toothpick obstruction and perforation has been previously observed to mimic other conditions of the gastrointestinal tract, presenting initially as conditions such as acute appendicitis and colonic diverticulitis, which ultimately were perforations of the terminal ileum and colon, respectively [13,14]. Toothpick perforation of the stomach, with accompanying ulcerating gastritis of unknown origin, has been documented [15]. However, to our knowledge, toothpick perforation of the terminal ileum, with stricture secondary to the presence of EAEC, has not been documented.

## Conclusions

EAEC creates conditions that set the stage for the necrosis of gastrointestinal epithelial tissue and mucosa ulceration. Perforation of the gastrointestinal tract by foreign bodies is not common as most foreign bodies pass through spontaneously. However, underlying pathologies can create strictures and other abnormalities in the gastrointestinal tract, which prevent foreign bodies from passing through.

There is limited literature on small bowel perforation and secondary peritonitis due to the ulceration of gastrointestinal mucosa by EAEC. Here, we report a case of EAEC leading to terminal ileum mucosal ulceration and complicated by perforation secondary to foreign body impaction associated with ileal stricture. The compounding effect of a foreign body such as a toothpick and mucosal ulceration caused by EAEC may explain the gastrointestinal tract perforation and secondary peritonitis.
